# Optical coherence tomography and multiphoton microscopy offer new options for the quantification of fibrotic aortic valve disease in ApoE^−/−^ mice

**DOI:** 10.1038/s41598-021-85142-4

**Published:** 2021-03-12

**Authors:** Anett Jannasch, Christian Schnabel, Roberta Galli, Saskia Faak, Petra Büttner, Claudia Dittfeld, Sems Malte Tugtekin, Edmund Koch, Klaus Matschke

**Affiliations:** 1 Department of Cardiac Surgery, Carl Gustav Carus Faculty of Medicine, Technische Universität Dresden, Heart Centre Dresden, Fetscherstraße 76, 01307 Dresden, Germany; 2 Department of Anesthesiology and Intensive Care Medicine and Clinical Sensoring and Monitoring, Carl Gustav Carus Faculty of Medicine, Technische Universität Dresden, Dresden, Germany; 3 Department of Cardiology, Heart Center Leipzig At University Leipzig, Leipzig, Germany

**Keywords:** Biomarkers, Cardiology, Diseases, Risk factors, Signs and symptoms, Optics and photonics

## Abstract

Aortic valve sclerosis is characterized as the thickening of the aortic valve without obstruction of the left ventricular outflow. It has a prevalence of 30% in people over 65 years old. Aortic valve sclerosis represents a cardiovascular risk marker because it may progress to moderate or severe aortic valve stenosis. Thus, the early recognition and management of aortic valve sclerosis are of cardinal importance. We examined the aortic valve geometry and structure from healthy C57Bl6 wild type and age-matched hyperlipidemic ApoE^−/−^ mice with aortic valve sclerosis using optical coherence tomography (OCT) and multiphoton microscopy (MPM) and compared results with histological analyses. Early fibrotic thickening, especially in the tip region of the native aortic valve leaflets from the ApoE^−/−^ mice, was detectable in a precise spatial resolution using OCT. Evaluation of the second harmonic generation signal using MPM demonstrated that collagen content decreased in all aortic valve leaflet regions in the ApoE^−/−^ mice. Lipid droplets and cholesterol crystals were detected using coherent anti-Stokes Raman scattering in the tissue from the ApoE^−/−^ mice. Here, we demonstrated that OCT and MPM, which are fast and precise contactless imaging approaches, are suitable for defining early morphological and structural alterations of sclerotic murine aortic valves.

## Introduction

Calcific aortic valve stenosis (CAVS) is the most common valvular heart disease in North America and European countries^1^ with a prevalence of 12.4% in people over 75 years old^2^. Currently, heart valve replacement is the only therapy available to treat people with CAVS and, thus 275,000 patients per year receive a transcatheter or surgical aortic valve replacement^3^. The early presentation of CAVS is aortic valve sclerosis (AVS), which involves fibrotic thickening of the aortic valve without hemodynamic changes^4^. The prevalence for AVS is about 30% in patients over 65 years old and up to 40% in patients over 75 years old^5,6^. It has been reported that 15% of patients with AVS develop mild to severe CAVS^7^. Gharacholou et al. defined AVS as an irregular non-uniform thickening of the aortic valve leaflets, commissures, or both with increased echogenicity, nonrestricted or minimally restricted opening of the aortic cusps, and a peak continuous-wave Doppler velocity across the valve ≤ 2 m/s^5^.

Lipid-lowering drugs, such as statins, have been used to treat CAVS, although randomized clinical trials^8–11^ showed that the drugs failed to stop disease progression or improve clinical outcomes^8–11^. It has been proposed that these trials failed because CAVS is considered to be a pathophysiological endpoint, while AVS is regarded as an intermediate phenotype that can benefit from statin application^12,13^. Although AVS is currently not considered to be a pathological condition and it typically depicts normal echocardiography findings, it is the pathological basis for processes such as dystrophic calcification and progress to CAVS. As up to now no reliable biomarkers for sclerotic changes in aortic valve tissue exist, there is a demand for emerging imaging techniques to quantify AVS^5,12^.

In clinical practice, transthoracic echocardiography is still the gold standard that is used to diagnose and estimate the severity of CAVS^14–16^. Studies have utilized animal models as tools to clarify the underlying mechanisms of the initiation^17^ and progression of CAVS^18^; mouse models are widely used^19–24^. Typically, echocardiography^19–23,25^ and histological examinations^19,26–29^ are employed to determine the pathological of the aortic valve. High-resolution echocardiography systems^30–32^ can be used to detect the early characteristics of AVS^20,23^, while the morphological valve structure is characterized histologically. Therefore, it is not possible to monitor the individual temporal progression from the early changes that occur in AVS to calcific aortic valve disease (CAVD).

Over the last decade, optical coherence tomography (OCT) has been used in a broad range of biomedical applications in clinical diagnosis and research^33–36^. OCT is an interferometric imaging technique using near-infrared light that offers a contact-free and marker-free visualization of tissue volume and morphology on a micrometer scale. Thus, OCT allows repetitive in vivo measurements in animal disease models or the clinical monitoring of patients without causing harm or side effects^33,36^.

Multiphoton microscopy (MPM) enables marker-free analysis of bulk tissue samples. In contrast with OCT, MPM provides images that are confocal and based on morphological and biochemical contrasts. Similar to OCT, it uses near-infrared lasers, which penetrate deep in the tissue and avoid photodamage. Tridimensional visualization is possible using MPM by acquiring stacks of images. MPM is commonly used to visualize fibrillar collagen by utilizing second harmonic generation (SHG) microscopy^37–39^. A direct quantification of collagen content^40^ is possible using SHG microscopy under controlled experimental conditions. SHG imaging of collagen has also been used to characterize heart valve diseases^41–43^, aortic^44^ and pulmonary artery^45^ dysfunction, and cardiac fibrosis^42,46–48^. Furthermore, the use of more complex systems, including two laser sources with an adapted wavelength and multiple detection units, enables multimodal MPM with the simultaneous generation and acquisition of SHG imaging together with coherent anti-Stokes Raman scattering (CARS). CARS is especially useful for the imaging of lipids^49^ and has also been used to visualize the pathological lipid accumulation in atherosclerotic plaques^50–52^ and in heart^53^ and valve diseases^43^.

In this study, OCT and MPM marker-free imaging approaches were used to define early structural alterations in the aortic valves of hyperlipidemic mice (ApoE^−/−^) compared with wild type (WT) mice. The focus was on leaflet thickening, as well as lipid and collagen content.

## Results

**Figure 1:**
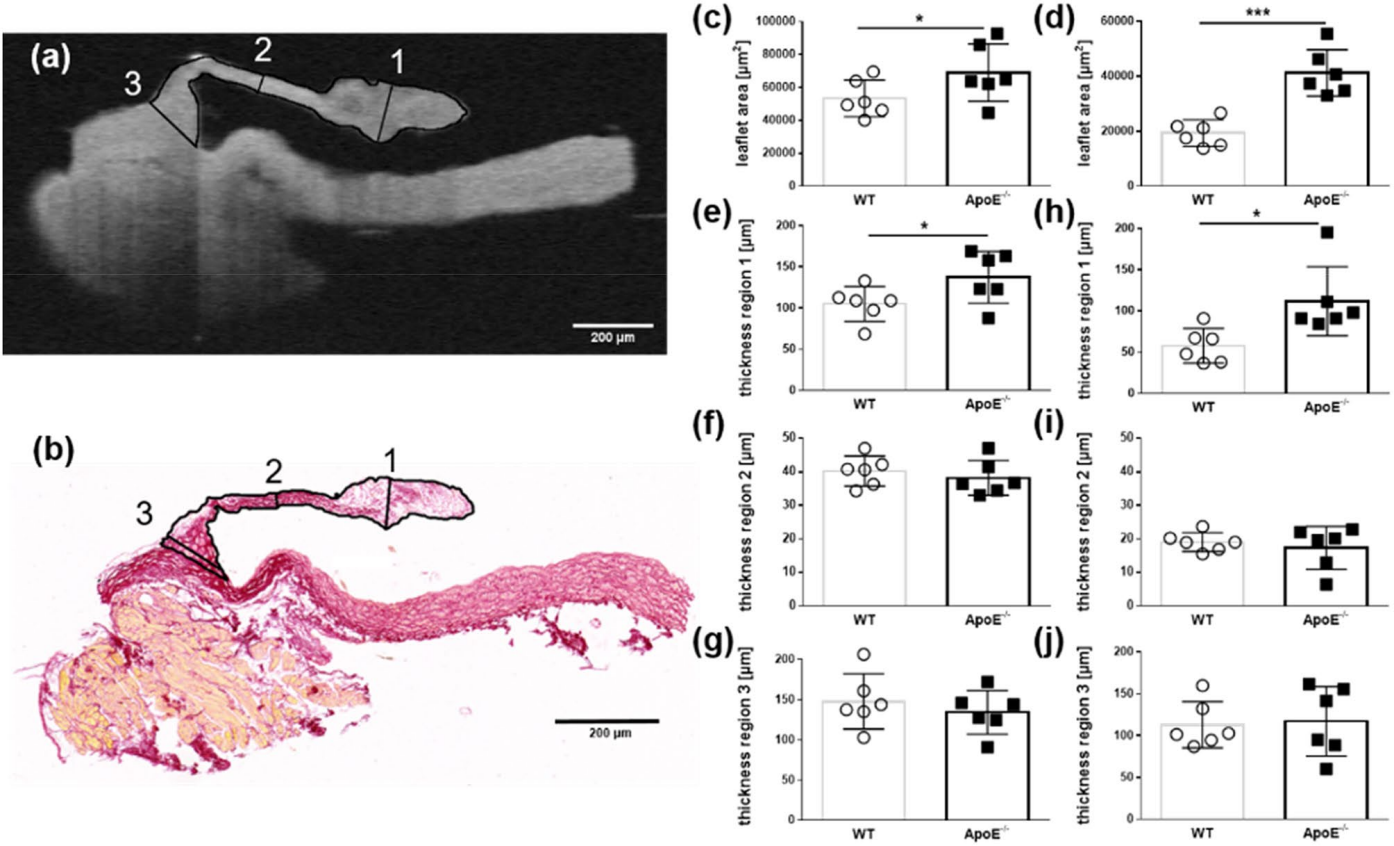
Quantification of the leaflet area and thickness in WT and ApoE^−/−^ mice (*n* = 6). (**a**) OCT scan of a representative acoronary aortic leaflet showing the allocation of the grid pattern to define regions 1, 2, and 3. (**b**) Histological section of the same representative acoronary aortic valve leaflet and corresponding regions. (**c**) The mean leaflet area and (**e–f**) thickness of the acoronary aortic valve leaflets from the OCT scans. (**d**) The mean leaflet area and (**h–j**) thickness of the acoronary aortic valve leaflets from histological sections. MW ± SD; *P ≤ 0.05, **P ≤ 0.01, and ***P ≤ 0.001.

The aortic valve leaflets were separated into three previously described regions for the characterization^26^. Region 1 encompassed the free edge of the tip of the leaflet (*nodulus valvulae semilunaris*). Region 2 covered the middle of the leaflet, while region 3 covered the base of the leaflet, which included the aortic root, but excluded the aortic wall (Fig. 1a,b).

### Determination of leaflet dimensions: OCT versus histological analysis

The OCT cross-sections and the histological sections of the valvular leaflets matched very well (Fig. 1a,b). Depending on the tissue processing and slicing procedures, histological sections can be hampered by structural changes. The analysis of the OCT scans revealed that the ApoE^−/−^ mice had a significantly increased mean leaflet area (69,003 µm^2^  ± 7,138 µm^2^) compared with the WT mice (53,386 µm^2^ ± 4,546 µm^2^, *P* = 0.047, Fig. 1c). In a subsequent histological analysis, the leaflet area of the ApoE^−/−^ mice compared with the WT mice was 41,264 µm^2^ ± 8,421 µm^2^ and 19,321 µm^2^ ± 4831 µm^2^, respectively (*P* = 0.0001, Fig. 1d). The OCT scans showed that the leaflet thickness in region 1 increased to 137 µm ± 31 µm in the ApoE^−/−^ mice from 105 µm ± 18 µm in the WT mice (*P* = 0.031, Fig. 1e). There were no differences in the average thickness in region 2 or region 3 between the ApoE^−/−^ mice and the WT mice (Fig. 1f/g, i/j). These findings correlated with the descriptions in the histological sections: the thickness of region 1 in the WT mice was wild type 58 µm ± 21 µm, while the thickness in the ApoE^−/−^ mice was 112 µm ± 42 µm (*P* = 0.013, Fig. 1h). The individual absolute area values of the histologically processed leaflets were found to be 2.4-fold smaller than the area values of the OCT processed native leaflets (Supplementary Table S1).

### Bleaching of the melanin

**Figure 2:**
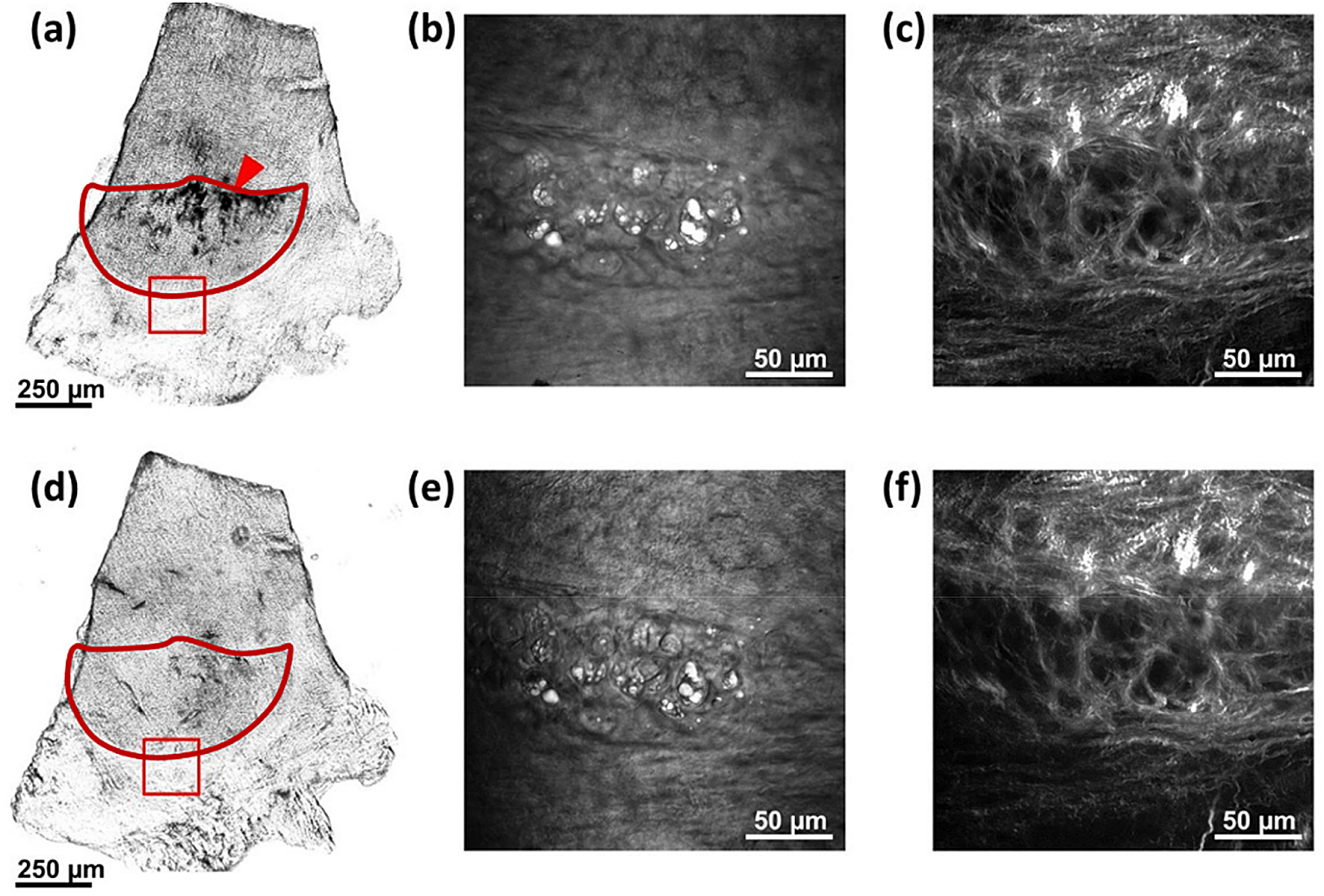
Images of a leaflet before and after melanin bleaching in 2.5% H_2_O_2_ solution. (**a**) Bright field image of the whole leaflet (red contour) before bleaching showing visible accumulations of melanin (red arrowhead). (**b**) CARS image of the region of the annulus in the red box in (**a**) acquired before bleaching with the adipocytes clearly identified by the intense CARS signal of the lipid droplets. (**c**) SHG image of the region in the red box in (**a**) acquired before bleaching showing the dense mesh of collagen fibers. (**d**) Bright field image of the whole leaflet (red contour) after bleaching without any visible melanin. (**e**) CARS image of the region in the red box in (**d**) acquired after bleaching with the adipocytes clearly identified by the intense CARS signal of the lipid droplets. (**f**) SHG image of the region in the red box in **(d)** showing no alterations of the mesh of collagen fibers after bleaching.

Murine heart valves contain melanocytes; the pigmentation level of murine heart valves may correlate with coat color^54^. Because of the genetic background, the aortic valve leaflets of C57BL/6 J WT mice and ApoE^−/−^ mice are pigmented. The melanin is highly efficient at absorbing light, including UV and near infrared light and, thus, hinders MPM by absorbing both the generated signals and the excitation laser light. The latter phenomenon often leads to strong photodamage, which can include burning of the pigmented tissue. For this reason, it was necessary to eliminate the melanin before imaging by bleaching the isolated and formalin-fixed valves in a hydrogen peroxide (H_2_O_2_) solution in water. This approach has been used for years for the histopathology of melanoma, although different protocols have been employed, which range from incubation in 10% H_2_O_2_ for 24 h at room temperature^55^ to incubation in 3% H_2_O_2_ for 2 h at 55°C^56^. As this method had not been tested for MPM of murine valves, we investigated incubation in different concentrations (1.25% to 15% H_2_O_2_) for 24 h at room temperature. Incubation in 2.5% H_2_O_2_ was found to remove all melanin without producing significant alterations in the SHG signal of the collagen fibers or the CARS signal of the lipids (Fig. 2). Thus, this protocol was used in all of the following experiments. We found that higher concentrations of H_2_O_2_ altered the tissue morphology, which was visible both in the SHG and the CARS images, while lower concentrations were not able to bleach the melanin completely.

### Collagen content

**Figure 3:**
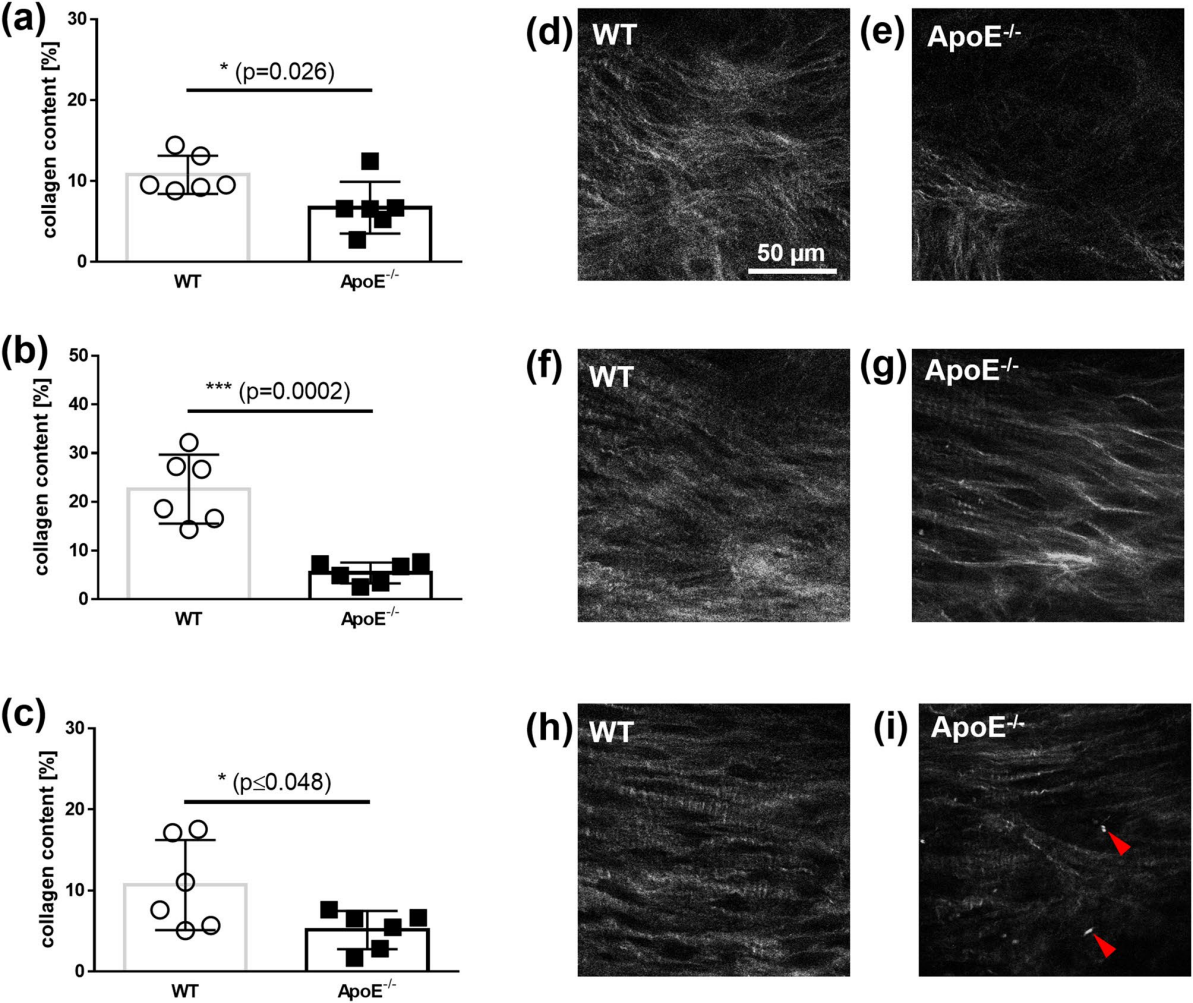
Quantitative analysis of the collagen content in a leaflet using SHG images. The mean collagen content from WT mice and ApoE^−/−^ mice from (**a**) the region 1; from (**b**) region 2; and from (**c**) the region 3. Representative SHG images of the region 1 of the aortic valve leaflets from (**d**) WT mice and (**e**) ApoE^−/−^ mice. Representative SHG images of the region 2 from (**f**) WT mice and (**g**) ApoE^−/−^ mice. Representative SHG images of the region 3 of (**h**) WT mice and (**i**) ApoE^−/−^ mice, acquired in the middle of the thickness. The dimensions of all images are 140 µm × 140 µm.

A semi-quantification of the amount of collagen in a leaflet was performed by analyzing stacks of SHG images. The mean relative amount of collagen in the ApoE^−/−^ mice compared with the WT mice is displayed in Fig. 3. The ApoE^−/−^ mice (*n* = 6) showed a significantly lower amount of collagen compared with the WT mice (*n* = 6) in region 1: 6.72% ± 3.19% versus 10.78% ± 2.37%, respectively (*P* = 0.026, Fig. 3a). The ApoE^−/−^ mice also had a significantly lower amount of collagen compared with the WT mice in regions 2 and 3: 5.42% ± 2.14% versus 22.62% ± 7.08%, respectively (*P* = 0.0002, Fig. 5b); and 5.13% ± 2.37% versus 10.68% ± 5.57%, respectively (*P* = 0.048, Fig. 3c). This is also shown in the images comparing the animals with the highest and lowest collagen amounts (Fig. 5d–i).

**Figure 4:**
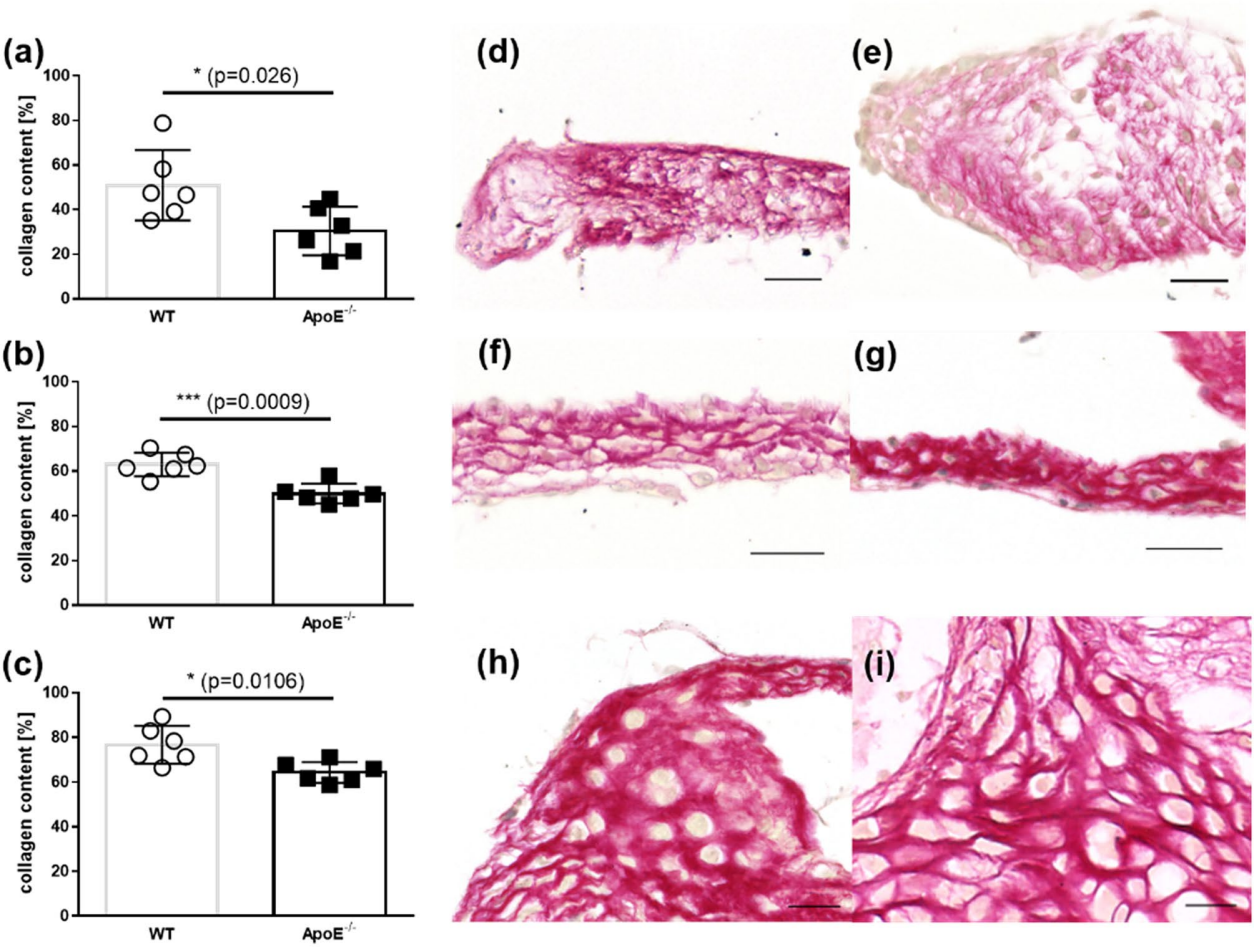
Quantitative analysis of picrosirius red-stained slides of aortic valve leaflets. The mean collagen content from (**a**) the region1, (**b**) the region 2, and (**c**) the region 3 from WT and ApoE^−/−^ mice (*n* = 6). Representative picrosirius red-stained slides of the region 1 from (**d**) WT mice and (**e**) ApoE^−/−^ mice, of the region 2 from (**f**) WT mice and (**g**) ApoE^−/−^ mice, and of the region 3 of (**h**) WT and (**i**) ApoE^−/−^ mice. The scale bar is 20 µm.

**Figure 5:**
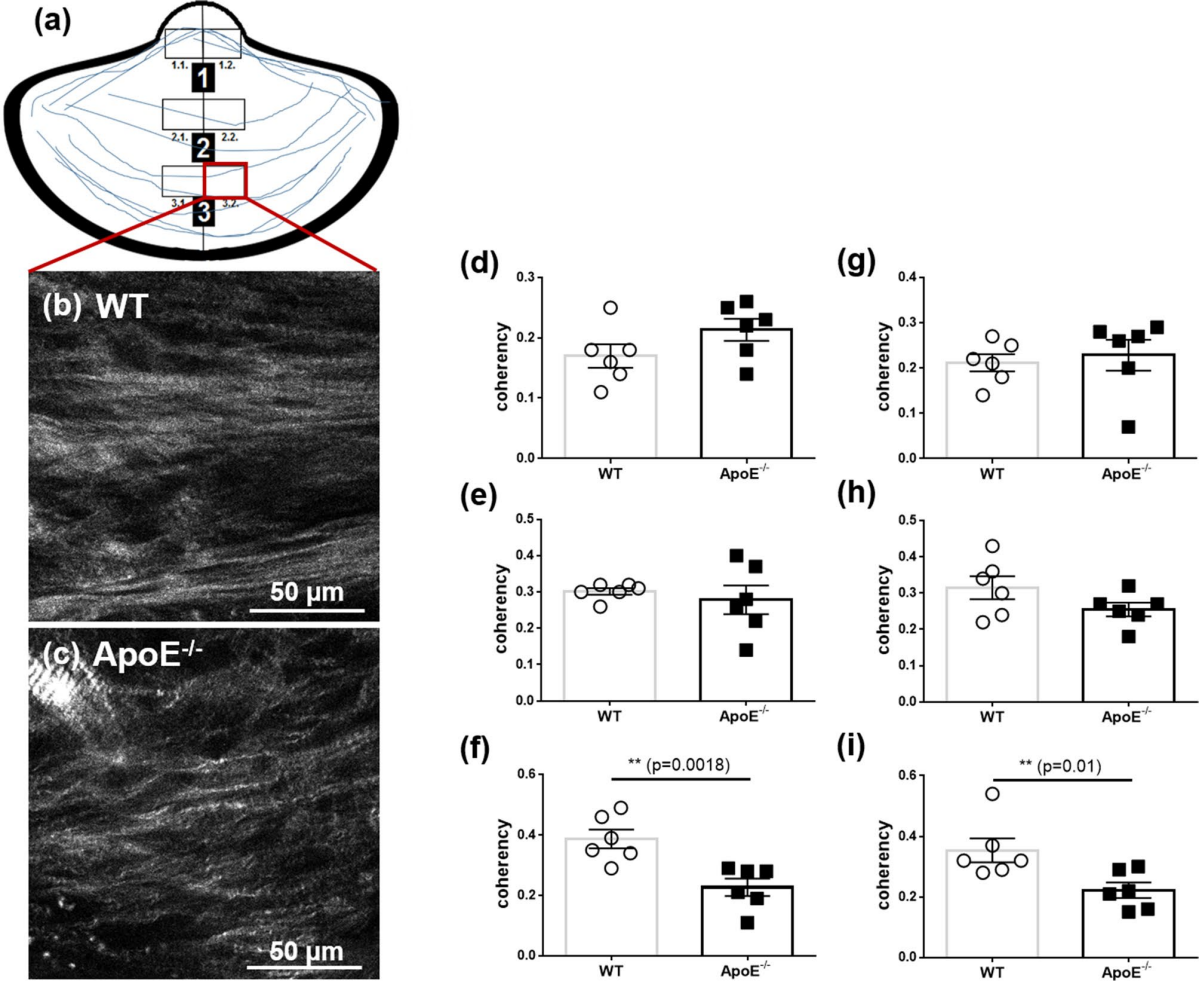
SHG images showing the coherency of collagen fibers. Orientation of collagen fibers (blue) and analyzed subregions within the tip (region1), middle (region 2) and base (region 3) of the aortic leaflet. Representative images from region 3.2. from (**b**) WT mice and (**c**) ApoE^−/−^ mice. The mean coherency of the collagen from (**d**) the region 1.1 (**g**) and the region 1.2, from (**e**) the region 2.1 and (**h**) the region 2.2, from (**f**) in the region 3.1 and (**i**) the region 3.2 from WT mice and ApoE^−/−^ mice.

The quantitative histological analysis of picrosirius red-stained sections revealed that the aortic valves of the ApoE^−/−^ mice (*n* = 6) contained significantly less collagen than the WT mice (*n* = 6) in region 1: 30.4% ± 10.9% versus 50.9% ± 15.8%, respectively (*P* = 0.026, Fig. 4a). This trend continued in regions 2 and 3: 50.0% ± 4.3% versus 63.0% ± 5.3% (*P* = 0.0009, Fig. 4b) and 64.4% ± 4.7% versus 76.8 ± 8.5%, respectively (*P* = 0.0106, Fig. 4c). Respective picrosirius red-stained pictures (Fig. 5d–i) also indicate significant less collagen in all observed regions of ApoE^−/−^ mice compared with WT mice.

### Collagen fiber orientation

The orientation of collagen in the WT mice and the ApoE^−/−^ mice is displayed in Fig. 5. The collagen structure appeared disordered in the ApoE^−/−^ mice (Fig. 5b) compared with WT mice (Fig. 5a). Because the orientation direction of collagen fibers is arranged circumferential to the annulus, images of aortic valve leaflets were divided in the middle resulting in the left and right halves (Fig. 5a subregions 1 and 2). The analysis revealed that there was a significantly lower coherency in base regions 3.1 and 3.2 in the ApoE^−/−^ mice (*n* = 6) compared with the WT mice (*n* = 6): 0.23 ± 0.03 versus 0.39 ± 0.03 (*P* = 0.0018, Fig. 5f) and 0.22 ± 0.03 versus 0.35 ± 0.04 (*P* = 0.0095, Fig. 5i), respectively. There was no difference in coherency between the WT mice and the ApoE^−/−^ mice within the region 1 and the region 2 of the leaflet structure (Fig. 5d, e and g, h).

### Lipid content

**Figure 6:**
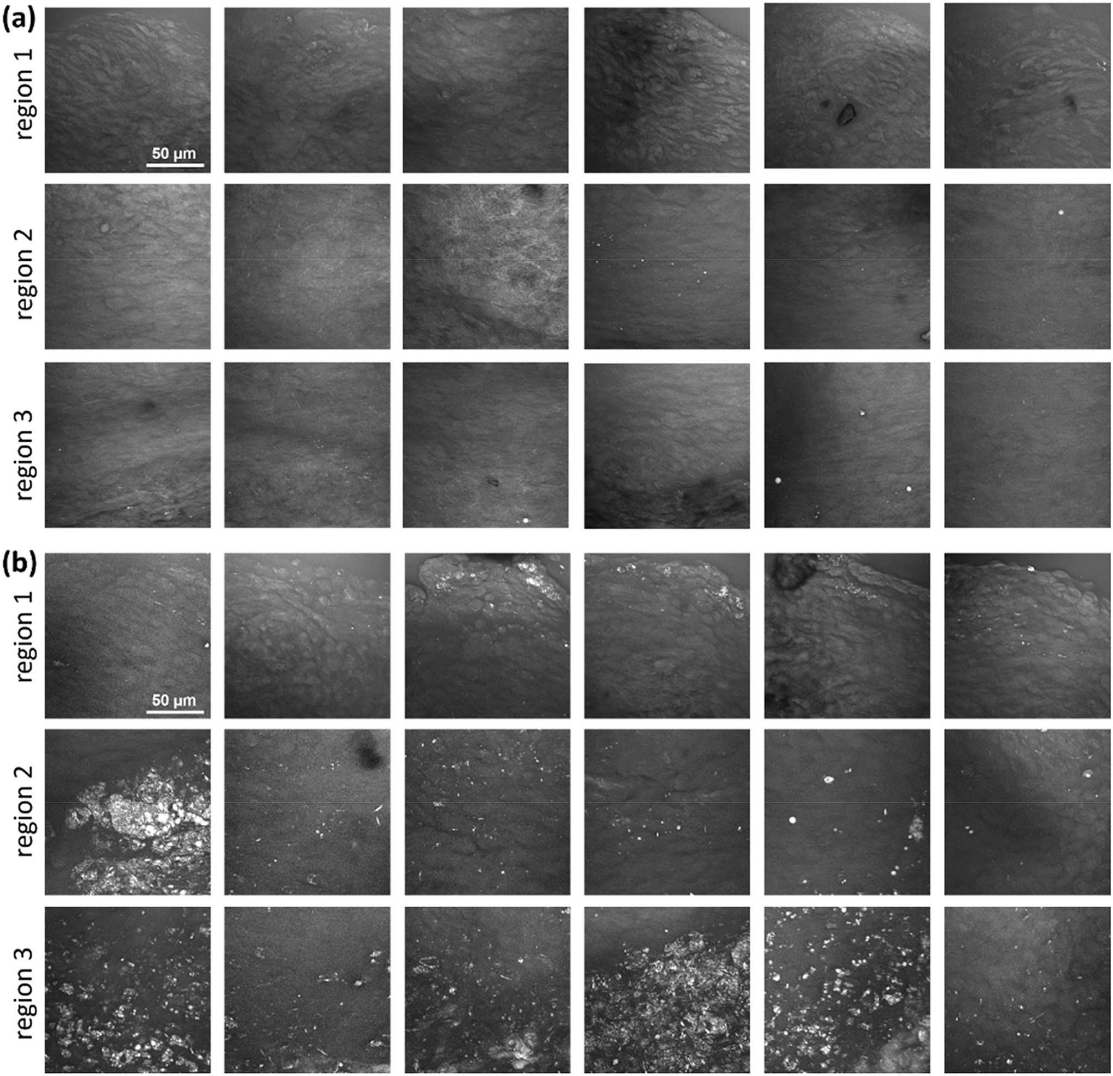
CARS images of acoronary leaflets acquired in the region 1, 2 and 3. (**a**) WT mice. (**b**) ApoE^−/−^ mice. All images are maximum intensity projections of the stacks acquired through the entire leaflet thickness and have the dimensions of 140 µm × 140 µm.

A qualitative evaluation of lipid accumulations in the form of cholesterol crystals and lipid droplets was performed using image stacks acquired using CARS. A maximum intensity projection that displays the total amount of lipids in the leaflet regions is shown in Fig. 6.

**Figure 7:**
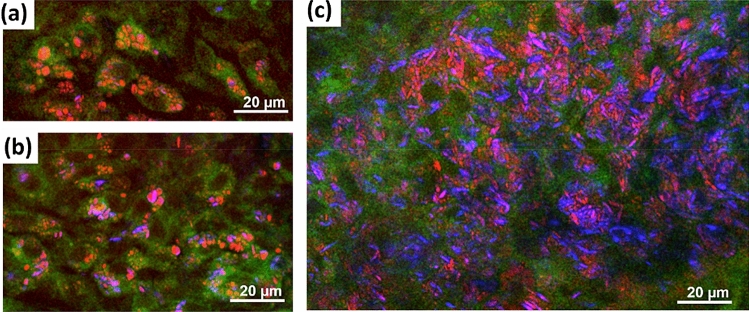
Multimodal images of acoronary leaflet regions of ApoE^−/−^ mice showing the morphology of lipid accumulations. Red channel: CARS; green channel: TPEF; blue channel: SHG. (**a**) Intracellular lipid droplets. (**b**) Intracellular lipid droplets and cholesterol crystals. (**c**) A massive accumulation of lipid and cholesterol crystals without clear intracellular localization. In all images, the green TPEF visualizes cytoplasm and the nucleus, which is devoid of fluorophores, appears as dark hole.

No lipids were found in the region 1 and 2 of all analyzed WT mice. Only sporadic and very small lipid droplets were observed in four of the six WT mice in the region 3 near the annulus. In contrast, the leaflets of the ApoE^−/−^ mice showed a massive accumulation of lipids in the form of cholesterol crystals and lipid droplets. All ApoE^−/−^ mice displayed lipid accumulation in the region 2 and region 3. Three of the six ApoE^−/−^ mice also showed lipid droplets in the region 1. From the morphology, lipid droplets often seemed associated with cells, which were identified as foam cells (Fig. 7a,b). Cholesterol crystals, identified by the crystal structures, were observed inside the cells (Fig. 7b), and accumulated in plaques within the tissue (Fig. 7c). In the latter case, a cytoplasmic or extracellular localization could not be determined from the multiphoton images.

## Discussion

Currently, the only treatment for CAVD is interventional or surgical valve replacement. Potential pharmacological interventions should target early alterations, such as AVS, although this would require the precise identification of disease onset and progression. Small animal models have been used in the development of experimental therapy approaches^8^. The current methods used for the visualization of aortic valve morphology and function in animal models are limited because of the inadequate resolution, the need for markers, and the excessive histological processing^24,57^. Although some molecular imaging^58^ and micro-computed tomography^59^ approaches offer increased resolution and the opportunity to study pathophysiological processes in vivo, these techniques are not broadly available and are associated with high costs.

OCT and MPM exhibit resolutions that are in the micrometer range; both techniques are contactless and do not use markers. OCT has been used in a study on the quantification of murine ventricular volume and mass^20^. We previously showed that 4D OCT high-speed video microscopy is suitable for measuring the valve opening area in an artificially stimulated heart model^60^. MPM has been used to characterize human explanted aortic valves^43,61^, cardiac valve allografts^62^, and tissue-engineered heart valves^63^ in which the intensity of the SHG signal was used to quantify the collagen fibers^42,61^. This is the first study that examined the aortic valve geometry and structure of C57Bl6 WT mice and age-matched ApoE^−/−^ mice using OCT and MPM. ApoE^−/−^ mice have been used in animal models to study AVS^18,64^. We found that fibrotic thickening, collagen reduction, and lipid accumulation were significantly different in the ApoE^−/−^ mice compared to WT mice using OCT and MPM.

The flexibility, shape, and surface structure of aortic valve leaflets are essential for the proper function and sufficient closure of the valve^65^. The base and tip regions of the leaflet are particularly prone to an instantaneous increase in tensile strength during the cardiac cycle^66^. Accordingly, we found an increase in the leaflet area and thickness of the leaflet tip in ApoE^−/−^ mice compared with WT mice using OCT; these results were confirmed by histology. These findings agreed with the histological studies of Aikawa et al.^64^ and Hinton et al.^26^ in which the transversal and sagittal directions, respectively, were chosen for sectioning. Comparing these studies with our observations showed that different directions of sections and tissue shrinkage from essential pretreatments present major limitations to histological examinations; these limitations make it difficult to rank results from different studies. Furthermore, many studies use transversal probes^27,67^, which are associated with the risk of overestimating the extent of the leaflet structures. The degree of tissue shrinkage in our study was 40–64% based on leaflet area, which is in accordance with the previously measured values of data on murine aortic valve tissue^26^. Our data demonstrated the superiority of OCT and an important refinement in determining leaflet thickening during the development of AVS in small animal models because it was possible to observe the native tissue in a fast, precise, and tridimensional fashion.

AVS is an actively regulated cellular process^68^ in which resident valvular interstitial cells differentiate into an activated myofibroblast state in response to an altered mechanical stress^69^ and pro-fibrotic cytokines. Myofibroblasts produce extracellular matrix components and remodeling enzymes^70^ that cause structural changes. In ApoE^−/−^ mice on a hypercholesterolemic diet, the thickening of valve structures is associated with the presence of macrophage-rich lesions^64^. It is well known that macrophages and activated valvular interstitial cells acquire excessive levels of proteolytic enzymes, such as collagenase-1 (MMP-1), collagenase-3 (MMP-13), gelatinase A (MMP-2), and gelatinase B(MMP-9), that contribute to collagen and elastin degradation and lead to valvular remodeling^71–73^.

In this study, we determined the collagen content by analyzing the acquired SHG signal in MPM and compared the results with the collagen content in equivalent histological sections. We found that the collagen content decreased in the aortic valves in ApoE^−/−^ mice compared with the WT mice in all three of the analyzed regions. This is in accordance with the work of Trapeaux et al. who demonstrated a decreased collagen content in the cross-sections of the aortic valves from 32-week-old ApoE^−/−^ mice that had received a high fat diet^74^. The study found that the reduction in collagen content was most pronounced in the middle region of the leaflet; this distribution pattern was confirmed in the current study. It should be noted that a lower amount of collagen was detected by MPM compared with the histological results. This may be explained by the fact that the amount of collagen was based on the fraction of sample area displaying the SHG signal or picrosirius red staining and by the inherent confocality of MPM. While the SHG signal was collected from a laser focus depth of about 2 µm, the staining signal was collected from the entire thickness of the slide by light transmission microscopy. We also studied the orientation of the collagen meshwork in the three analyzed regions and found less orientation in the base region of the ApoE^−/−^ mice. This agreed with a study by Chu et al.^23^ who found an altered orientation of collagen fibers in a hypercholesterolemic/hypertensive mice model at the base of the cusps. They assumed that, although the total collagen content in the valve was not increased or further reduced, the remodeling of the collagen contributed to the restricted valve opening. It needs to be mentioned that Chu and coworkers could only describe the orientation of the base on transversal histological sections. Using the SHG imaging, we demonstrated an analysis of collagen fibers over the entire valve dimension. This allows a more detailed analysis of the collagen meshwork.

Lipid accumulation represents another hallmark of early aortic valve lesions^75^. It is desirable to use pharmacological interventions that target lipid accumulation before there is evidence that fibrocalcific aortic valve disease has developed^57^. One study showed that the accumulation of oxidized phospholipids to large cholesterol crystal structures in mice fed a high cholesterol diet was involved in the inflammatory processes within aortic valve disease^76^. We denoted an increase in both the lipid content and the cholesterol crystal content in the base and middle regions of the leaflets in ApoE^−/−^ mice, but not in WT mice. It is noteworthy that non-crystalline cholesterol particles are not detected by SHG signals because only non-centrosymmetric and crystalline structures are captured^77,78^. Chu et al. demonstrated that there were increased levels of lipids in the aortic valves from elderly ApoE^−/−^ mice^23^. Although we did no lipid staining, we detected more pronounced holes, which resulted from cut adipocytes and lipid particles, in the middle region and especially in the annulus region of the leaflet from ApoE^−/−^ mice. This observation is in accordance with the respective results of CARS signal analysis.

Our data demonstrated that OCT and MPM are suitable for quantifying early pathomechanisms in aortic valve disease in a murine model, such as fibrotic thickening and fatty degradation. OCT and MPM showed collagen reduction, lipid accumulation, and thickening of the aortic valve structure, which correlated with the results from conventional histological examinations. We concluded that the implementation of OCT and MPM has great potential in the early diagnosis of CAVD in murine models.

### Limitations

The bleaching process, necessary before MPM, made the valve tissue porous and hindered cryosectioning. Therefore the murine aortic valves were embedded in paraffin for the histological analysis and lipid staining was not possible.

## Methods

### Animals and tissue processing

Five-week-old female ApoE^−/−^ (B6.129P2-Apoe^tm1Unc^/J) mice were purchased from Charles River Laboratories (Sulzfeld, Germany). Following 1 week of acclimatization, the mice were fed a Western-type diet (TD88137 mod.; Ssniff, Soest, Germany), which contained 21.2% total fat, 2.071 mg/kg cholesterol, and 17.5% protein for 16 weeks. Aged-matched female C57BL/6JRj mice, purchased from Janvier Labs (Le Genest-Saint-Isle, France), served as controls and received a standard diet (V1534 R/M-H; Ssniff, Soest, Germany) for 16 weeks. After the feeding period, the mice were sacrificed by cervical dislocation, and the ascending aorta, including the aortic valves, was excised. The aortic valve structure was opened using a longitudinal incision between the right and left coronary leaflet. This preparation was used to visualize native, non-fixed aortic valve tissue under ex vivo conditions with OCT, as described in a previous study^60^. Prior to the MPM scans, the aortic valve tissue was fixed in 4% formaldehyde and bleached after incubation in 2.5% H_2_O_2_ for 24 h at room temperature. Subsequently the aortic valve tissue was embedded in paraffin for histological analysis (see Supplementary Fig. S1).

The animal research ethics committee of the Technische Universität Dresden and the Regional Council (Regierungspräsidium) Dresden approved the experiment according to the institutional guidelines and the German animal welfare regulations (AZ: 24-9168.24-1/2013-10).

### Optical coherence tomography (OCT)

The OCT system used in this study is a custom-made frequency-domain spectrometer-based device with a 12-kHz A-scan rate and a lateral and axial resolution of 11 µm and 6.4 µm, respectively as described in detail in a previous study^60^.

### OCT data processing and analysis

The open-source software Fiji^79^ was used for all image processing and analyses. In OCT, the lateral and axial resolution are not directly linked. While the axial resolution depends on the used wavelength, bandwidth, and the spectrometer design, the lateral resolution depends on beam shaping and focusing. Therefore, the first step in image processing involved scaling the image data stack to achieve uniform voxel sizes in all three dimensions. Furthermore, as an optical imaging modality, OCT measures the optical path length. For the measurement of geometric lengths, such as leaflet thickness, the optical refractive index of the tissue must be taken into account. Although the exact refractive index of aortic valve tissue is not known. Since the heart valves generally correspond to an endothelial duplication, the refractive index could be in the range of 1.33 to 1.44, but nothing is known about the change of refractive index due to the different age, diet and stage of disease in wildtype and ApoE knockout mice. Any assumption about this lead to additional inaccuracies in thickness measurements.

**Figure 8:**
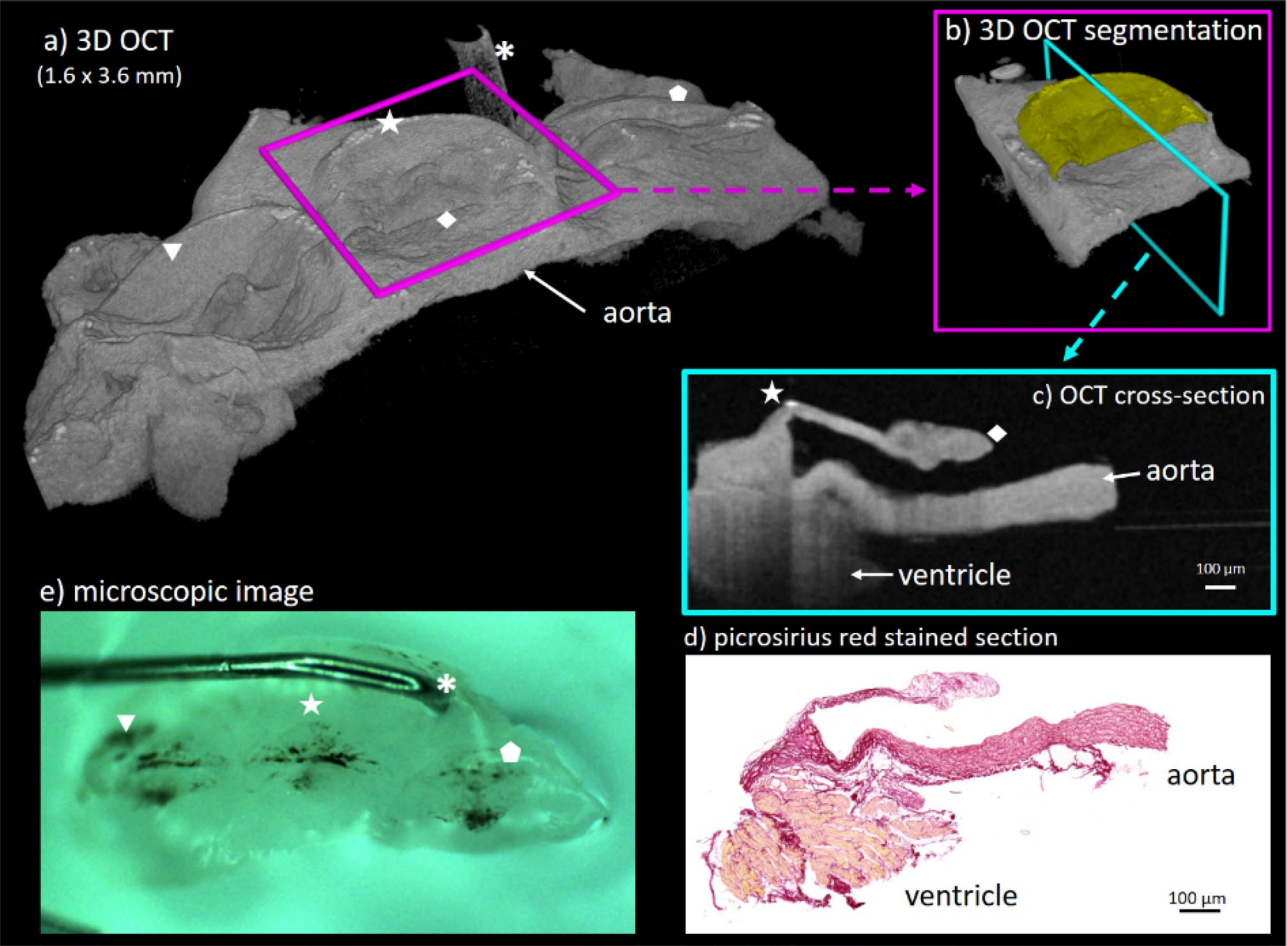
OCT visualization of an ex vivo aortic valve after tissue processing. The tridimensional OCT data stack enables a wide range of possibilities for image processing, including volumetric visualization, segmentation, and cross-sectional views. The OCT cross-sections were chosen to be perpendicular to the base of the acoronary leaflet. In contrast with the histological slides, OCT can be applied to native tissue and cross-sections in any orientation and, thus, can be used for tissue characterization and measurements. (**a**) 3D OCT scan of the right coronary (triangle), acoronary (star) and left coronary leaflet (pentagon). Further image processing and measurements were performed on acoronary leaflet only (**b**) and (**c**)). The base of the acoronary leaflet is marked with the star and the tip of the nodulus is marked with the rhombus. To further support visual image correlation, the asterisk (**a**) and (**e**)) marks the pin used to keep tissue in place.

Therefore, we assumed a mean refractive index of 1.33 (water) because all thickness measurements were compared relative to each other. After scaling, the voxel size was determined to be 2.255 µm in each direction. The tissue thickness was measured in a cross-sectional view perpendicular from the base to the tip (Fig. 8). To enhance the tissue contrast and the signal-to-noise ratio, the intensity values of ten adjacent cross-sections were summed. The leaflet area was measured by manual segmentation in the same cross-section.

### Multiphoton microscopy (MPM)

The MPM system has been described in detail elsewhere^80^. In short, the MPM system consists of 2 picosecond lasers emitting at 781 nm and 1,005 nm coupled to a laser scanning microscope. The SHG signal generated by the first laser can be detected in transmission mode with a band-pass filter centered at 390 nm and a bandwidth of 18 nm. The CARS signal of the symmetric stretching vibration of methylene groups, which corresponded to the Raman band at 2,850 cm^−1^, was detected in transmission mode using a band-pass filter centered at 647 nm and a bandwidth of 57 nm. Additionally, the two-photon excited fluorescence (TPEF) signal was acquired in the 500–550 nm range in the reflection configuration, which worked as a reference tissue morphology by visualizing the cellular structures. The excitation laser beams were focused with a C-Apochromat 32 × /0.85 objective.

For imaging, the isolated and prepared aortic valve was placed in a drop of phosphate-buffered saline between two coverslips with the leaflet up and the aorta below. Stacks of images were acquired from the tip, the middle region, and the base of the right posterior/acoronary leaflet (Fig. 1a and b) for the subsequent evaluation of the collagen and lipid content from the SHG and CARS images, respectively. Each stack of images spanned the entire thickness of the leaflet and was composed of 14 images; the z-step was varied depending on the local thickness of each leaflet in order to acquire only the leaflet and not the underlying aorta. The same acquisition parameters, such as laser power and photomultiplier gain, a pixel dimension of 0.28 µm, and 65,536 Gy levels (16-bit image), were used for all images.

### MPM image analysis

**Figure 9:**
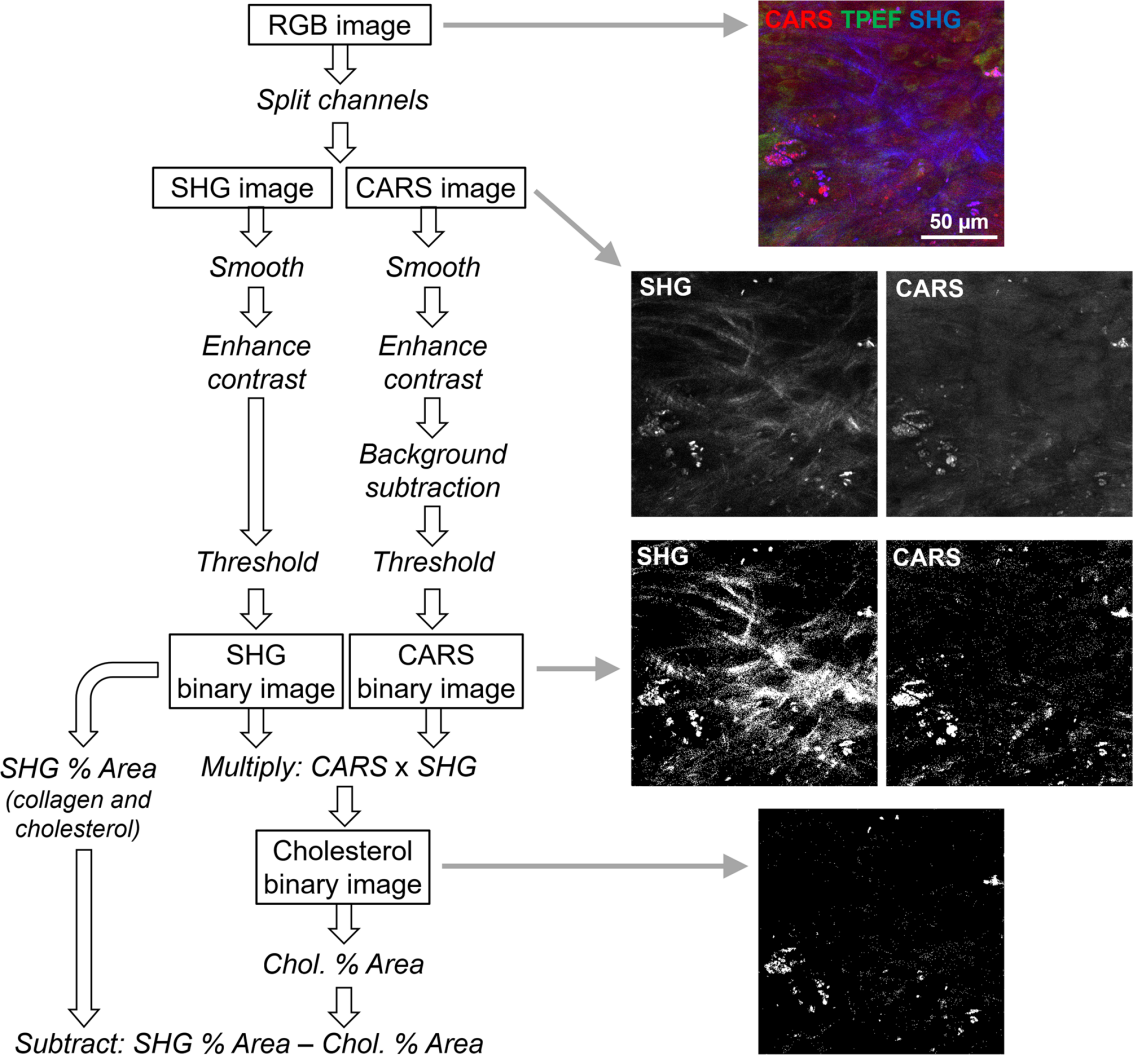
Flow-chart of the image analysis procedure used for collagen quantification in MPM images.

The open-source software Fiji^79^ was used to analyze all images. The collagen content was based on the percentage of tissue area that had a SHG signal. First, a mean filter with a radius of 1 pixel was used to smooth the SHG images; this filter smooths the image by replacing each pixel with the neighborhood mean, where the size of the neighborhood is specified by the radius in pixels. Smoothing was followed by a linear contrast enhancement (min–max with 0.4% saturated pixels). The area in the image with a signal intensity above a threshold of 20,000 was measured and used to determine the fraction of tissue containing collagen; the original images of a representative subset were used to define the threshold value. A visual inspection of the binary images took place after thresholding. The procedure was applied to all the images in each stack and was used to calculate the percentage fraction of the area with a SHG signal for each image. However, this area was not always representative of the collagen content because the cholesterol crystals also had a SHG signal. Therefore, a correction was performed to subtract the contribution of the cholesterol crystals, which was based on the fact that the crystals also generated intense CARS signal. For this purpose, all lipid structures were identified on the CARS images. First, a mean filter with a radius of 1 pixel was used to smooth the CARS images, followed by a linear contrast enhancement (min–max with 0.4% saturated pixels). A background subtraction was performed using a radius of 30 pixels. A threshold was then applied to create a binary image using the triangle method for automatic threshold setting. Finally, the binary CARS images were multiplied with the binary SHG images. In this way, only the structures that were both SHG-active and CARS-active (i.e., the cholesterol crystals) were retained. The percentage fraction of the area was then measured and subtracted from the percentage fraction of the area with the SHG signal for each image. A flow-chart of the whole analysis procedure with example images is displayed in Fig. 9. Finally, a mean value for each leaflet region was obtained by averaging the percentage fraction of the area with the corrected SHG signal of all the images in the stack. The degree of organization of collagen fibers was retrieved using the plugin OrientationJ for ImageJ. The coherency parameter was used to quantify the orientation order of the fiber^81^. The coherency in an image has a value of one if the fibers are perfectly oriented in one direction and a value of zero for an isotropic structure without any orientation. Because the orientation direction of collagen fibers was different in the two halves of images, which were acquired from the midline of the leaflet, OrientationJ was applied to the left and right halves of the images (subregions 1 and 2).

### Histological staining

The paraffin-embedded aortic structures containing the aortic valves were sectioned at 3 µm in the longitudinal direction and stained with Movat’s pentachrome and picrosirius red (see the Supplementary Information). Movat’s pentachrome stain was used according to the manufacturer’s instructions (Kit 12,061; Morphisto GmbH, Frankfurt, Germany). All stained sections were acquired using a Zeiss Axio Scan.Z1 slide scanner (magnification 10 ×) and were manually segmented to determine the leaflet area and thickness using ZEN 3.1 software (Carl Zeiss Microscopy GmbH, Jena, Germany).

### Measurement of leaflet area and leaflet thickness and assessment of collagen content in histological samples

The acoronary aortic leaflets (*valvula semilunaris posterior*) were used for all histological analyses. The section with the maximum dimension of leaflet structure was determined after careful evaluation of the serial sections for each sample. The leaflet area and thickness were manually segmented using ZEN 3.1. software (Carl Zeiss Microscopy GmbH, Jena, Germany). The leaflet thickness was determined using a grid pattern in the three regions^26^: Region 1 was at the free edge of the tip (*nodulus valvulae semilunaris*); region 2 was the middle of the leaflet and region 3 was at the hinge or the base of the semilunar valve and included the aortic root, but excluded the aortic wall. A line was scattered perpendicular to the leaflet surface in the above mentioned three regions (Fig. 1a,b). The total collagen content was assessed in the positive picrosirius red-stained sections using the ImageJ plugin Colour Deconvolution and a manually defined threshold^82^. Data represented the percentage of valve area that displayed positive staining.

### Statistics

Data were presented as the means ± standard deviation (SD) and the normal distribution was tested with the Kolmogorov–Smirnov test. The Student’s unpaired one-tailed *t*-test was used to compare the two experimental groups when the data had a normal distribution; otherwise, the Mann–Whitney U test was employed. *P*-values < 0.05 were considered to be significant. GraphPad Prism 6.0 software (GraphPad Software, Inc., La Jolla, USA) was used for the statistical analysis and visualization.

## Supplementary Information

Supplementary Information

## Data Availability

The data that support the findings of this study are available from the corresponding author upon reasonable request.
