# Supplementary material for: Optical coherence tomography and multiphoton microscopy offer new options for the quantification of fibrotic aortic valve disease in ApoE−/− mice
Source: Sci Rep. 2021 Mar 12;11:5834. doi: 10.1038/s41598-021-85142-4 (PMC7955095; doi:10.1038/s41598-021-85142-4)
Supplement: Supplementary file 1 — Supplementary Information [file 41598_2021_85142_MOESM1_ESM.docx]

**Supplementary Information**

**“Optical coherence tomography and multiphoton microscopy offer new options for the quantification of fibrotic aortic valve disease in ApoE^–/–^ mice”**

Anett Jannasch^1*+^, Christian Schnabel^2+^, Roberta Galli^2+^, Saskia Faak^1^, Petra Büttner^3^, Claudia Dittfeld^1^, Sems Malte Tugtekin^1^, Edmund Koch^2^, Klaus Matschke^1^

^1^ Department of Cardiac Surgery, Carl Gustav Carus Faculty of Medicine, Technische Universität Dresden, Heart Centre Dresden, Germany

^2^ Department of Anesthesiology and Intensive Care Medicine and Clinical Sensoring and Monitoring, Carl Gustav Carus Faculty of Medicine, Technische Universität Dresden, Germany

^3^ Heart Center Leipzig at University Leipzig, Department of Cardiology, Leipzig, Germany

^*^ correspond to Dr. Anett Jannasch e-mail: anett.jannasch@tu-dresden.de

**Supplementary Methods**

**Embedding orientation and section alignment**

The aortic valve structure was fixed in formaldehyde in a horizontal orientation following OCT and MPM, and was embedded in paraffin in an upright position. The section direction was radial and perpendicular to the aortic cusp surface and the aortic valve leaflets were successively cut in 3 µm thin sections. Supplementary Figure S1 illustrates the direction in which the tissue was sectioned and the resulting aortic leaflet structures.

**Picrosirius red staining**

The 3-µm paraffin sections were deparaffinized, rehydrated, and stained with picrosirius red for 1 h. The stain was prepared using 0.5 g sirius red (#365548, Sigma-Aldrich Chemie GmbH, Taufkirchen, Germany) in 500 ml picric acid (P6744, Sigma-Aldrich Chemie GmbH, Taufkirchen, Germany) in 1.3% water. The sections were then dehydrated through an ascending series of ethanol and placed in xylene before being coverslipped with DePex (#18243, Serva Electrophoresis, Heidelberg, Germany).

**Supplementary Table**

Supplementary Table S1: The mean leaflet area and thickness in regions 1, 2, and 3 of the OCT processed (native) and histologically processed (histo) acoronary aortic leaflet.

|  | **Area [µm^2^]** | **thickness region1** | **thickness region 2** | **thickness region 3** |
| --- | --- | --- | --- | --- |
| **WT native** (n) | 53386.0±10165.9 (6) | 105.0±19.3 (6) | 40.2±4.1 (6) | 147.9±31.3 (6) |
| **WT histo** (n) | 19320.6±4409.7 (6)**** | 57.9±19.2 (6)** | 19.0± 2.6 (6)**** | 113.1±25.2 (6)* |
| **ApoE^-/-^** **native** (n) | 69003.0±15962.1 (6) | 137.4±28.7 (6) | 38.2±4.8 (6)* | 134.3±24.7 (6) |
| **ApoE^-/-^ histo** (n) | 41333.7±7653.9 (6)** | 111.9±38.3 (6) | 17.3±5.8 (6)**** | 117.1±37.8 (6) |

MW ± SD, Student’s unpaired one-tailed *t*-test , WT versus ApoE^–/–^, * p ≤ 0.05, ** p ≤ 0.01, *** p ≤ 0.001, and **** p ≤ 0.0001

**Supplementary Figure**


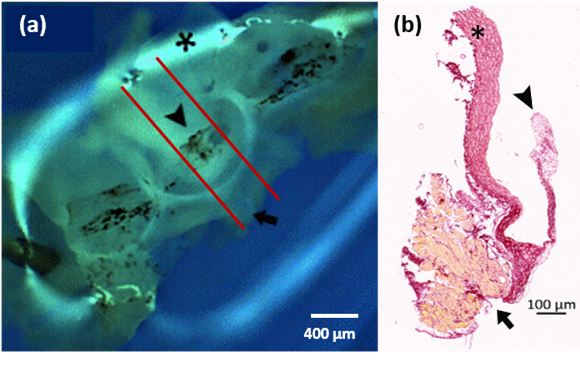
Supplementary Figure S1. **(a)** The bright field image of a murine aortic valve preparation illustrating the orientation of the specimen; the aortic ring was opened between the right and left coronary cusp and pinned in a horizontal position. Note that the red line highlights the selected specimen area. **(b)** The side view of a histological picrosirius red-stained section of a non-coronary aortic valve leaflet. Symbols: star = aorta ascendens, triangle = aortic valve leaflet, and arrow = ventricle.
